# Antiulcer Potential of *Olea europea* L. cv. Arbequina Leaf Extract Supported by Metabolic Profiling and Molecular Docking

**DOI:** 10.3390/antiox10050644

**Published:** 2021-04-22

**Authors:** Arafa Musa, Nourhan Hisham Shady, Shaimaa R. Ahmed, Taghreed S. Alnusaire, Ahmed M. Sayed, Bassam F. Alowaiesh, Ibrahim Sabouni, Mohammad M. Al-Sanea, Ehab M. Mostafa, Khayrya A. Youssif, Dalia H. Abu-Baih, Mahmoud A. Elrehany, Usama Ramadan Abdelmohsen

**Affiliations:** 1 Pharmacognosy Department, College of Pharmacy, Jouf University, Sakaka, Aljouf 72341, Saudi Arabia; emmoustafa@ju.edu.sa; 2 Department of Pharmacognosy, Faculty of Pharmacy, Al-Azhar University, Cairo 11371, Egypt; 3 Department of Pharmacognosy, Faculty of Pharmacy, Deraya University, New Minia City, Minia 61111, Egypt; Norhan.shady@deraya.edu.eg; 4 Department of Pharmacognosy, Faculty of Pharmacy, Cairo University, Kasr El-Aini Street, Cairo 11562, Egypt; shaimaa.ahmed@pharma.cu.edu.eg; 5 Department of Pharmacognosy, College of Pharmacy, Jouf University, Sakaka, Aljouf 2014, Saudi Arabia; 6 Biology Department, College of Science, Jouf University, Sakaka, Aljouf 72341, Saudi Arabia; tasalnosairi@ju.edu.sa (T.S.A.); bfalawish@ju.edu.sa (B.F.A.); 7 Department of Pharmacognosy, Faculty of Pharmacy, Nahda University, Beni-Suef 62513, Egypt; Ahmed.mohamed.sayed@nub.edu.eg; 8 Olive Research Center, Jouf University, Sakaka, Aljouf 72341, Saudi Arabia; ibrahimsabouni@gmail.com; 9 Pharmaceutical Chemistry Department, College of Pharmacy, Jouf University, Sakaka, Aljouf 72341, Saudi Arabia; mmalsanea@ju.edu.sa; 10 Department of Pharmacognosy, Faculty of Pharmacy, Modern University for Technology and Information, Cairo 11371, Egypt; Khayrya.youssif@pharm.mti.edu.eg; 11 Department of Biochemistry and molecular biology, Faculty of Pharmacy, Deraya University, New Minia City, Minia 61111, Egypt; Dalia.hamdy@deraya.edu.eg (D.H.A.-B.); Mahmoud.elrehany@deraya.edu.eg (M.A.E.); 12 Department of Biochemistry, Faculty of Medicine, Minia University, Minia 61519, Egypt; 13 Department of Pharmacognosy, Faculty of Pharmacy, Minia University, Minia 61519, Egypt

**Keywords:** *Olea europea*, metabolic profiling, antioxidant, gastro-protective, 5-lipoxygenase

## Abstract

Gastric ulceration is among the most serious humanpublic health problems. *Olea europea* L. cv. Arbequina is one of the numerous olive varieties which have scarcely been studied. The reported antioxidant and anti-inflammatory potential of the olive plant make it a potential prophylactic natural product against gastric ulcers. Consequently, the main goal of this study is to investigate the gastroprotective effect of *Olea europea* L. cv. Arbequina leaf extract. LC-HRMS-based metabolic profiling of the alcoholic extract of *Olea europea* L. cv. Arbequina led to the dereplication of 18 putative compounds (**1**–**18**). In vivo indomethacin-induced gastric ulcer in a rat model was established and the *Olea europea* extract was tested at a dose of 300 mg kg^−1^ compared to cimetidine (100 mg kg^−1^). The assessment of gastric mucosal lesions and histopathology of gastric tissue was done. It has been proved that *Olea europea* significantly decreased the ulcer index and protected the mucosa from lesions. The antioxidant potential of the extract was evaluated using three in vitro assays, H_2_O_2_ scavenging, xanthine oxidase inhibitory, and superoxide radical scavenging activities and showed promising activities. Moreover, an in silico based study was performed on the putatively dereplicated compounds, which highlighted that 3-hydroxy tyrosol (**4**) and oleacein (**18**) can target the 5-lipoxygenase enzyme (5-LOX) as a protective mechanism against the pathogenesis of ulceration. Upon experimental validation, both compounds 3-hydroxy tyrosol (HT) and oleacein (OC) (**4** and **18**, respectively) exhibited a significant in vitro 5-LOX inhibitory activity with IC_50_ values of 8.6 and 5.8 µg/mL, respectively. The present study suggested a possible implication of *O. europea* leaves as a potential candidate having gastroprotective, antioxidant, and 5-LOX inhibitory activity for the management of gastric ulcers.

## 1. Introduction

Mediterranean people frequently used olive leaf trees in the treatment of several diseases [1]. Olive leaves exert a wide range of ethnopharmacological activities such as antimicrobial, immunomodulatory, and anti-inflammatory properties [2]. Several biological applications were exhibited by *Olea europea* such as its effect as a diuretic, laxative, antiviral, and hypotensive agent [3]. Moreover, it has been reported for the management of intestinal and stomach diseases, as well as diabetes [3,4,5].

Previously, Dekanski et al. [6] have been reported that the *Olea europea* leaf extract was effective in the reduction of gastric lipid peroxidation and in maintaining the natural antioxidative enzyme activity. These results have indicated that the *Olea europea* leaf extract has a promising gastroprotective potential against ethanol-induced gastric lesions in rats. Moreover, a previous report has shown that oleuropein, the main constituent of olive leaves extract, was able to exert a potent antioxidant potential and gastroprotective effect [7].

Gastritis is one of the health problems which is characterized by inflammation of the lining epithelial layer of the stomach [8]. Moreover, permanent gastritis in some cases is considered a pre-cancer lesion [9]. This serous medical condition could lead to severe pain in the abdomen as well as, heartburn, which could be accompanied by bleeding, and various gastrointestinal complications [8].

Non-steroidal anti-inflammatory drugs (NSAIDs) are among the most common causes behind this medical condition, where they are able to disrupt the cyclooxygenase-dependent-gastro-protection. At the same time, they activate the 5-lipoxygenase (5-LOX) signaling pathway that eventually led to gastric mucosal injury due to the elevated oxidative stress and severe vasoconstriction [10,11,12,13]. In addition to inhibiting cyclooxygenase and decreasing prostaglandin production, NSAIDs induce mucosal damage via ROS production by recruited leukocytes. ROS-mediated mitochondrial damage as well as lipid, protein, and DNA oxidation lead to apoptosis and mucosal injury [12,13].

Traditionally, olive leaves were used to cure many different ailments and reported to exert a gastro-protective activity [8]. According to Mahdavi et al. [14], the *Olea europea* extract can decrease the side effects of indomethacin on the intestinal tissue and enhances the gastrointestinal function, and hence, it could be considered as a potential herbal supplement in the treatment of intestinal morphological injuries. The treatment with the *Olea europea* extract was able to reduce the ulcer index and the total stomach acidity which were induced by the administration of high doses of indomethacin. It was also able to decrease both the gastric juice volume and the acid pepsin secretion, in addition to the protection of mucosa by gastric mucin activity [15]. In another investigation, oleuropein has been found to exert intestinal anti-inflammatory, antioxidant, and anti-apoptotic effects in the ulcerative colitis experimental model [16].

**Figure 1:**
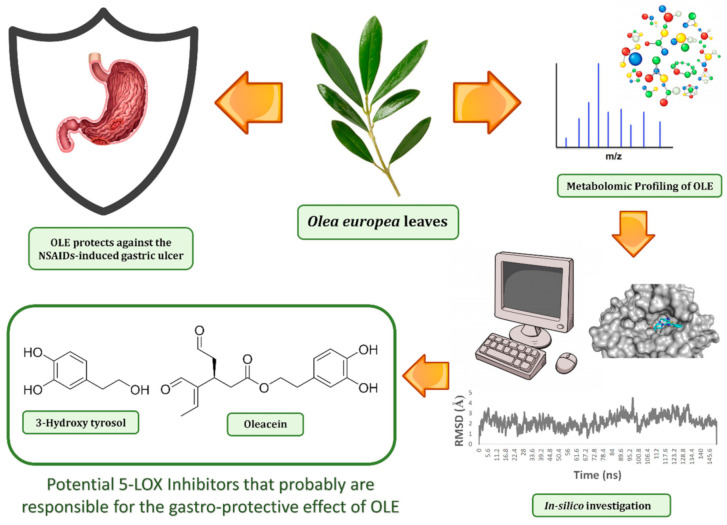
The workflow applied in the present study.

Consequently, these interesting findings provoked us to investigate *Olea europea* L. cv. The Arbequina leaves extract (OLE) for its antiulcer potential both in vivo and in vitro with the aid of several in silico techniques (e.g., pharmacophore-based virtual screening, ensemble docking, and molecular dynamics simulation to determine the main biological active constituents in OLE (Figure 1).

## 2. Material and Methods

### 2.1. Plants Material

The leaves of *Olea europea* L. cv. Arbequina were collected in May 2020 from the Bosita area, Jouf, Saudi Arabia. It was authenticated by Mr. Hamedan Al-Ogereef, Camel and Range Research Center, Jouf, KSA. A voucher specimen (2020-BuPD 76) was archived at the Pharmacognosy Department, Faculty of Pharmacy, Beni-Suef University, Egypt.

### 2.2. Preparation of the Extract

Until exhaustion, dried powder of *Olea europea* (4 kg) was extracted with ethanol (3 × 10 L). The dried residue (210 g) was then obtained by the evaporation of ethanol extract under reduced pressure.

### 2.3. Metabolic Profiling

LC-MS was carried out using a Synapt G2 HDMS quadrupole time-of-flight hybrid mass spectrometer (Waters, Milford, CT, USA). The sample (2 μL) was injected into a BEH C18 column (2.1 × 50 mm), which was adjusted to 40 °C, and connected to the guard column. The gradient elution of mobile phase was used, the mobile phase consisted of purified water (A) and acetonitrile (B) with 0.1% formic acid in each solvent. The gradient program started with 10% B and B was increased linearly in 30 min to 100% B at a flow rate of 300 µL/min and remained isocratic for 5 min before linearly decreasing in 1 min to 10% B. The column was then re-equilibrated with 10% B for 9 min before the next injection. The total analysis time for each sample was 45 min. The injection volume was 10 µL and the tray temperature was maintained at 12 °C. High resolution mass spectrometry was carried out in both positive and negative ESI ionization modes with a spray voltage of 4.5 kV and capillary temperature of 320 °C. The mass range was acquired from *m*/*z* 150 to 1500. The MZmine 2.12 was employed for differential investigation of MS data, followed by converting the raw data into positive and negative files in mzML format with ProteoWizard. The compounds were then dereplicated using the Dictionary of Natural Products (DNP) database.

### 2.4. In Vitro Antioxidant Activity

#### 2.4.1. H_2_O_2_ Scavenging Activity

The determination of H_2_O_2_ scavenging activity that reflects the anti-oxidative capacity of the extracts was performed by the reaction with a defined amount of exogenously provided hydrogen peroxide. The antioxidants in the sample eliminate a certain amount of the provided hydrogen peroxide. The residual hydrogen peroxide was determined colorimetrically [17]. Briefly, 20 µL of the sample was added to 500 µL of hydrogen peroxide and incubated for 10 min at 37 °C. Later, 500 µL of enzyme/3, 5-dichloro-2-hydroxyl benzensulfonate mixture was added and incubated for 5 min at 37 °C. The intensity of the colored product was measured colorimetrically at a wavelength of 510 nm. The percentage of hydrogen peroxide scavenging activity was calculated by comparing the results of the test with those of the control using the following formula: H_2_O_2_ scavenging activity = {(A control − A sample)/A control) × 100}.

The IC_50_ value of each sample was calculated after performing the assay at four different concentrations, using the Graph pad prism 7 software.

#### 2.4.2. Superoxide Radical Scavenging Activity

The superoxide anion scavenging activity was measured as previously described [18]. The superoxide anion radicals were generated in a Tris–HCL buffer (16 mM, pH 8.0), containing 90 µL of NBT (0.3 mM), 90 µL NADH (0.936 mM) solution, 0.1 mL extract of different concentrations (125, 250, 500, 1000 μg/mL), and 0.8 mL Tris–HCl buffer (16 mM, PH 8.0). The reaction was started by adding a 0.1 mL PMS solution (0.12 mM) to the mixture, incubated at 25 °C for 5 min and the absorbance was measured at 560 nm against a blank, ascorbic acid was used as a reference. The percentage inhibition was calculated by comparing the results of the test with those of the control using the formula.

Superoxide scavenging activity = {(A control − A sample)/A control) × 100}.

The IC_50_ value was calculated using the Graph pad prism 7 software by performing the test at four different concentrations.

### 2.5. In Vivo Antiulcer Potential

#### 2.5.1. Experimental Design

The study was carried out on a population of adult male albino rats divided into four groups with each group consisting of six rats (200 ± 50 g) concerning all the guidelines of the National Institutes of Health for the care and the proper use of animals in the laboratory. Rats were deprived for 24 h of food before the experiments but we allowed the free use of water to make sure that the stomach was empty. Moreover, rats were placed in mesh-bottomed cages to dimension the coprophagia. Furthermore, all rats were adapted to the surrounding environment for 7 days before the beginning of our study to decrease any factor that may cause animal suffering. Finally, all of the animals were tested in our study at the same time of the day to exclude any variation that can lead to diurnal rhythms of putative regulators of gastric functions [19]. The total extract of *Olea europea* leaves was examined for its antiulcer potential using the indomethacin-induced gastric ulcer model in rats [19,20,21]. The experimental rats were classified randomly into four groups (i.e., six rats in each group). We give the treatment orally according to the following regimens: Group 1 served as the normal control group, which received the vehicle orally only (0.5% carboxymethyl cellulose (CMC)) only. Group 2 served as the positive control group and received the vehicle (0.5% CMC). Group 3 served as the market comparable reference group and was given oral cimetidine (100 mg/kg). Group 4 was administered the total extract at a dose level of 300 mg/kg p.o. (dissolved in a 0.5% CMC solution). After 1 h, groups 2–4 received a large dose of indomethacin (40 mg/kg) orally to induce gastric ulcers. After 4 h, all of the rats were sacrificed by cervical dislocation. Then, all of the stomachs were removed, incised along their greater curvature, followed by washing carefully with tap water, then with normal saline to get rid of all the gastric contents, followed by the examination for macroscopical mucosal lesions, and finally, the preparation for histopathological examination [20].

#### 2.5.2. Assessment of Gastric Mucosal Lesions

The ulcer index (UI) was calculated by examination of the stomachs with the aid of an eyepiece using a 0–3 scoring system. The severity factor is calculated as level 0 = no lesions, level 1 = lesions < 1 mm length, 2 = lesions 2–4 mm length, and 3 = lesions > 4 mm length. The lesions score for each rat was calculated as the number of lesions multiplied by their respective severity factor. The preventive index (PI) of a given drug was calculated by the following equation [22]:

(1) $$\mathrm{PI}=\frac{\left(\mathrm{UI}\;\mathrm{of}\;\mathrm{indomethacin}\;\mathrm{group}-\;\mathrm{UI}\;\mathrm{OF}\;\mathrm{OLE}-\mathrm{treated}\;\mathrm{group}\right)\times 100\;}{\left(\mathrm{UI}\;\mathrm{of}\;\mathrm{indomethacin}\;\mathrm{group}\right)}$$

#### 2.5.3. Histopathological Examination

A longitudinal gastric section was dissected from the glandular part of the stomach of each rat. Sections were fixed in a 10% buffered formalin solution, embedded in paraffin, and then sectioned at 4–5 mm using a microtome. Finally, paraffin was removed and the sections were stained with alum-hematoxylin and eosin and evaluated microscopically for histopathological changes. The images were captured using a LEICA, DM1000 microscope with a digital camera (LEICA, EC3, LEICA microsystems, Heppenheim, Germany).

### 2.6. Statistical Analysis

Data were expressed as the mean ± standard error of the mean (SEM) (*n* = 5). The one-way analysis of variance (ANOVA) followed by Dunnett’s test was applied. Graph Pad Prism 7 was used for statistical calculations (Graph pad Software, San Diego, CA, USA). Results were regarded significant as follows: * *p* < 0.05, ** *p* < 0.01, *** *p* < 0.001.

### 2.7. In Silico Based Molecular Target Identification

To find out the most probable molecular target for the main metabolites present in OLE, we used a web-based pharmacophore-based virtual screening tool namely, similarity ensemble approach (SEA) [23]. The smile codes of each compound were submitted to the server, and the retrieved molecular targets were arranged according to their probability values (*p*-value). The 5-lipoxygenase enzyme (5-LOX) is the top-scoring molecular target relevant to peptic ulcer pathogenesis that was predicted for both **4** and **18** (*p*-values = 6.232 × 10^−31^, and 6.654232 × 10^−21^, respectively). Docking experiments were performed using the AutoDock Vina software. The 5-LOX crystal structure with PDB codes of 6N2W was used for docking experiments. The applied docking protocol considers the protein as a rigid structure and the tested ligand as a flexible scaffold during its computations. The enzyme’s active site used for docking experiments was located according to the co-crystalized inhibitor (nordihydroguaiaretic acid; NDGA) [24], where we set the grid box of docking to enclose the part of the enzyme that was complexed with this co-crystalized ligand. The ligand-to-binding site shape matching root means square (RMSD) cutoff was set to 2.0 Å. The interaction energies were determined using the Charmm Force Field (v.1.02) with 10.0 Å as a non-bonded cutoff distance and distance-dependent dielectric. The tested compounds were energy-minimized inside the selected binding pocket. The editing and visualization of the generated binding poses were performed using the Pymol software [25].

### 2.8. Lipoxygenase (LOXs) Inhibition Assay

The potential inhibitory activity of the putative compounds **4** and **18** against the 5-LOX enzyme was investigated using human recombinant enzyme assay kits (catalog no. 60402 Cayman Chemical, Ann Arbor, MI, USA) following the manufacturer’s specifications. Stock solutions were freshly prepared and a buffer solution (0.1 M Tris HCl, PH, 7.4) was used. In addition, 10 µL of each compound was prepared (dissolved in the least amount of DMSO) and diluted with the stock solution to be in concentrations of (0.001, 0.1, 1, 5, 10 µM) to a final volume of 210 µL. The kinetic parameters for 5-LOX were determined by measuring the increase in absorbance at 238 nm using an Agilent 8453 Diode Array Spectrophotometer (Agilent Technologies, Santa Clara, CA, USA). The substrate concentration (5, 10, 20, 30, 40, 50 µM) was monitored in triplicate for each sample.

### 2.9. Docking Analysis and Molecular Dynamic Simulation Refinement

Molecular dynamic simulations (MDS) for ligand enzyme complexes were performed using the Nanoscale Molecular Dynamics (NAMD) 2.6 software [25], applying the CHARMM27 force field [26]. Hydrogen atoms were added to the protein structures using the psfgen plugin included in the Visual Molecular Dynamic (VMD) 1.9 software [27]. Afterwards, the whole generated systems were solvated using water molecules (TIP3P) and 0.15 M NaCl. At first, the total energy of the generated systems was minimized and gradually heated to reach 300 K and equilibrated for 200 s. Subsequently, the MDS was continued for 50 ns, and the trajectory was stored every 0.1 ns and further analyzed with the VMD 1.9 software. The MDS output was sampled every 0.1 ns to calculate the root mean square deviation (RMSD). The parameters of the tested compounds **4** and **18** were prepared using the online software Ligand Reader and Modeler (http://www.charmm-gui.org/?doc=input/ligandrm, accessed on 14 Janurary 2021) [27] and the VMD Force Field Toolkit (ffTK) [27]. Binding free energies were calculated using the free energy perturbation (FEP) method through the web-based software Absolute Ligand Binder [28] and the NAMD software.

## 3. Results and Discussion

### 3.1. Metabolic Profiling

**Figure 2:**
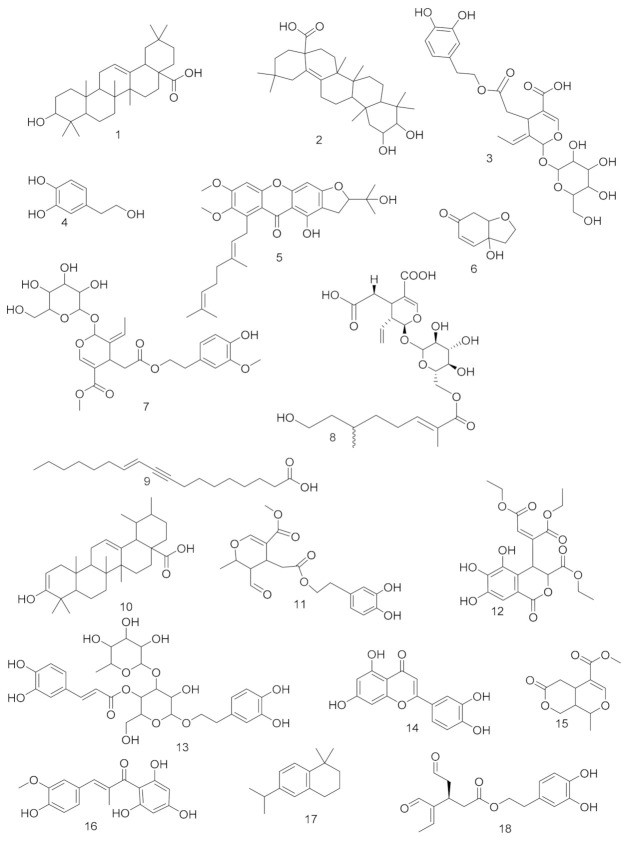
Putative compounds annotated from high-resolution mass-spectra data sets of *Olea europea*.

LC-MS profiling using HR-LCMS for the crude extract of *Olea europea* leaves was carried out to identify the chemical compounds responsible for the antiulcer potential. The putative dereplicated compounds shown in (Table S1, Figures S1 and S2; Figure 2) belonged to different phytochemical classes. Annotation of the putative compounds was carried out depending on HR-ESI-MS and a comparison with the data reported in the literature as 3-hydroxy-12-oleanen-28-oic acid; 3β-form (**1**) [29], 2,3-dihydroxy-13(18)-oleanen-28-oic acid; (2α,3β)-form (**2**) [30], oleuropein (**3**) [31], 2-(3,4-dihydroxyphenyl)ethanol (**4**) [32], oliverixanthone (**5**) [33], cleroindicin F (**6**) [34], oleuropein; 3″-Me ether (**7**) [35], oleoside (**8**) [35], 11-octadecen-9-ynoic acid; (E)-form (**9**) [36], 3-hydroxy-12-ursen-28-oic acid; 3β-form, 3-ketone (**10**) [37], 8-epimer, (3,4-dihydroxyphenylethyl) ester (**11**) [38], chebulic acid 4,5-didehydro(E-), tri-Et ester (**12**) [39], verbascoside (**13**) [40], luteolin (**14**) [41], olenoside A (**15**) [42], olivin (**16**) [6], olivacene (**17**) [43], and oleacein (**18**) [44].

### 3.2. In Vitro Antioxidant Activity

#### 3.2.1. H_2_O_2_ Scavenging Activity

**Figure 3:**
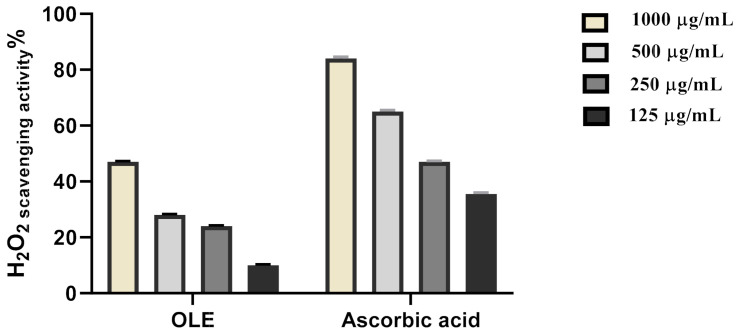
H_2_O_2_ radical scavenging activity of OLE ethanolic extract.

Scavenging of hydrogen peroxide is an important antioxidant activity. In this context, we evaluated the potential hydrogen peroxide scavenging activity of OLE. The maximum hydrogen peroxide radical scavenging activity for OLE was 47% at 1000 μg/mL concentration. OLE significantly inhibited the generation of hydrogen peroxide radicals in a dose-dependent manner showing a reliable antioxidant activity with IC_50_ of 279.2 μg/mL concentration for OLE (Figure 3, Table 1) and was compared with the standard ascorbic acid (IC_50_ = 178.7 μg/mL). This scavenging potential may be attributed to the phenolic content of the extract. NSAIDs are known to induce gastric ulcers via suppressing the cellular antioxidant enzymes and thus elevating the ROS level. The elevated ROS can attack cell membrane phospholipids as well as oxidatively damaging DNA repair mechanisms and proteins that regulate gene expression leading to altered cellular proliferation and apoptosis. These actions subsequently lead to epithelial damage, gastric mucosal damage, and ultimately gastric ulcer [45]. Hence, suppressing ROS levels via the administration of OLE may be the most relevant protective mechanism against NSAID-induced gastric ulcers. Previous studies showed that oleuropein which was the main active constituent in *Olea europea* can counteract oxidative stress and inflammation. It causes a significant reduction in colon MDA, MPO, and NO levels and a significant elevation in SOD, CAT, and GPX levels and caused downregulation of proinflammatory cytokines (IL-1β, TNF-α, IL-10, COX-2, iNOS, TGF-β1, MCP-1, and NF-κB) [16].

#### 3.2.2. Superoxide Radical Scavenging Activity

**Figure 4:**
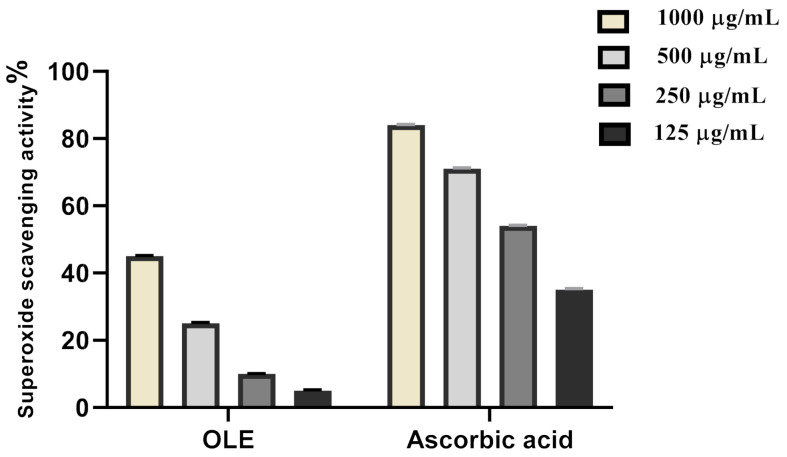
Superoxide radical scavenging activity of OLE ethanolic extract.

Over the past years, it has been elucidated that multiple reactive oxygen species are present in the biological systems that have the potential to induce damage. These reactive oxygen species include superoxide radical which participates in the formation of gastric lesions [46]. Therefore, it is rational to evaluate the superoxide scavenging activity of OLE and the results were shown in Figure 4 and Table 2. The scavenging effect increases with the concentration of standard and extract, the *Olea europea* extract exhibited the highest superoxide radical scavenging activity. At 1000 μg/mL concentration, the OLE ethanolic extract possessed 45% scavenging activity on superoxide. The concentration of OLE extract needed for 50% inhibition (IC_50_) was found to be 419.5 µg/mL, whereas 161.4 μg/mL was needed for ascorbic acid. This leads to the assumption that OLE is a potent gastroprotective by scavenging both hydrogen peroxide and superoxide radicals, as well.

### 3.3. In Vivo Antiulcer Potential

#### 3.3.1. Assessment of Gastric Mucosal Lesions

**Figure 5:**
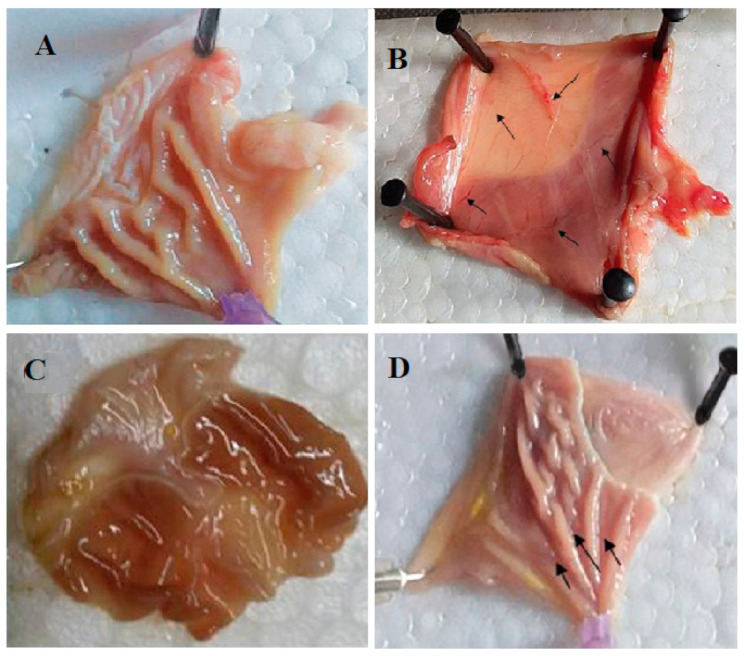
(**A**–**D**) Photos of stomachs of rats from different groups. (**A**) (Normal), (**B**) (negative control), (**C**) (cimetidine), (**D**) (total extract). Effect of OLE on the severity of the gastric lesion (gross examination) examined in the indomethacin-induced gastric ulceration model. (**A**) Control: Intact gastric mucosa tissues; (**B**) indomethacin (ulcer): Severe lesions are seen with extensive visible hemorrhagic necrosis of gastric mucosa; (**C**) cimetidine treated rats: Mild lesions of the gastric mucosa are observed compared to the lesions in indomethacin-induced ulcer rats; (**D**) OLE treated rats: Nearly normal gastric mucosa tissues. These photographs are typical of such tissues.

Investigation of the antiulcer potential of the crude methanolic extract showed that the total extract is a potent antiulcer extract with an ulcer index of 2.31 ± 2.17 and 98.0% inhibition of ulcer, relative to the cimetidine with 2.0 ± 1.01 ulcer index (Figure 5, Table 3). Many studies indicated that the gastroprotective potential of *Olea europea* leaves extract results from the ability of its constituents to scavenge reactive oxygen species, which initiate lipid peroxidation. The actual potential is probably related to its ability to maintain the integrity of the cell membrane, by its anti-lipid peroxidative activity and to protect in this way the gastric mucosa against oxidative damage, and by its ability to strengthen the mucosal barrier, the first line of defense against exogenous and endogenous ulcerogenic agents [47].

#### 3.3.2. Histopathological Examination

**Figure 6:**
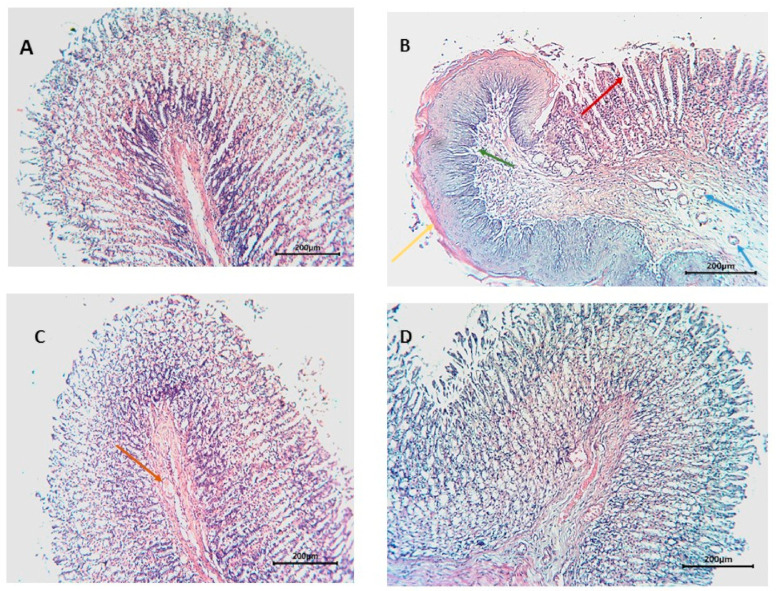
Histopathological examination of sections from stomachs of different groups. (**A**) (Normal), (**B**) (positive control), (**C**) (cimetidine), (**D**) (OLE).

The histopathological investigation of the normal structure of the gastric tissue consists of four layers: Lamina propria (L.P.), muscularis mucosa (M.m.), submucosa (S.m.), and muscularis externa (M.e.) [48]. Rats of the negative control group sections showed distortion of gastric tissue, extensive areas of hemorrhage, and thick mucus covering the surface (yellow arrows), moreover, necrosis to the muscularis externa (green arrows), as well as deformation of the fundic glands and chief cells (blue arrows), and finally cellular infiltration (red arrows). Cimetidine-treated groups exhibited wide areas of gastric pits, few edemas accompanied with few congested blood vessels (red arrows). The total extract-treated group showed mild necrotic lesions (red arrows) (Figure 6).

### 3.4. In Silico Based Molecular Target Identification

Characterization of the biological target for a given drug molecule is considered a challenging step in the process of drug discovery. However, the continuous development in the field of computer-aided molecular modeling and virtual screening has significantly facilitated this process. Many online target identification search engines are currently available, and their search protocols are either ligand-based or structural-based [49,50,51]. The similarity ensemble approach (SEA) is one of these online webservers that can screen and predict the potential protein targets of a query compound based on its pharmacophore model [23]. The concept of pharmacophore-based virtual screening is that the binding of certain drug molecules with their protein targets is mainly determined by key pharmacophore features (i.e., spatial arrangement of structural features). Hence, molecules that their scaffold can fit with these pharmacophore features have the potential to bind to the same molecular target. Consequently, SEA was used to propose a proper peptic ulcer-related molecular target (s) for the main OLE’s metabolites. Among the retrieved hits for each compound, we found that the 5-lipoxygenase enzyme (5-LOX) is the more molecular target relevant to peptic ulcer pathogenesis. Only compounds **4** and **18** were the putative compounds predicted to inhibit this enzyme, *p*-values = 6.232 × 10^−31^ and 6.654 × 10^−21^, respectively. Several previous reports have shown the pivotal role of 5-LOX and its produced signaling metabolites (i.e., leukotrienes) in the development of different types of gastric mucosal lesions [52], particularly those developed as a result of non-steroidal anti-inflammatory drugs (NSAIDs) misuse, where the inhibition of the cyclooxygenases (COX-1 and 2) abolish the gastro-protection and vasodilatory effect induced by prostaglandin E2 and prostacyclin (PGE2 and PGI2, respectively). Subsequently, such inhibition directs arachidonate to the 5-LOX pathway causing more vasoconstrictor 5-LOX products to accumulate, which in turn leads to a high tendency of mucosal damage particularly with augmented oxidative stress (Figure 5) [10]. Hence, 5-LOX inhibitors and leukotrienes inhibitors have been shown a significant protective effect against the gastric damage induced by noxious agents, particularly NSAIDs [10,11,12,13,52]. Consequently, we selected compounds **4** and **18** (i.e., 3-hydroxy tyrosol and oleacein, respectively) to be tested in vitro against 5-LOX as a validation step.

### 3.5. The 5-LOX Inhibition Assay

To validate the primary virtual screening prediction, both compounds 3-hydroxy tyrosol (HT) and oleacein (OC) (**4** and **18,** respectively) were assayed for their 5-LOX inhibitory activity with IC_50_ values of 18.6 and 5.8 µg/mL.

Previously, HT has shown potent inhibitory activity towards the production of leukotriene B_4_ by leukocytes [53]. Moreover, OC has been reported to produce 90% inhibition to the 5-LOX activity at a concentration of 10 µM [54].

### 3.6. Docking Analysis and Molecular Dynamic Simulation Refinement

**Figure 7:**
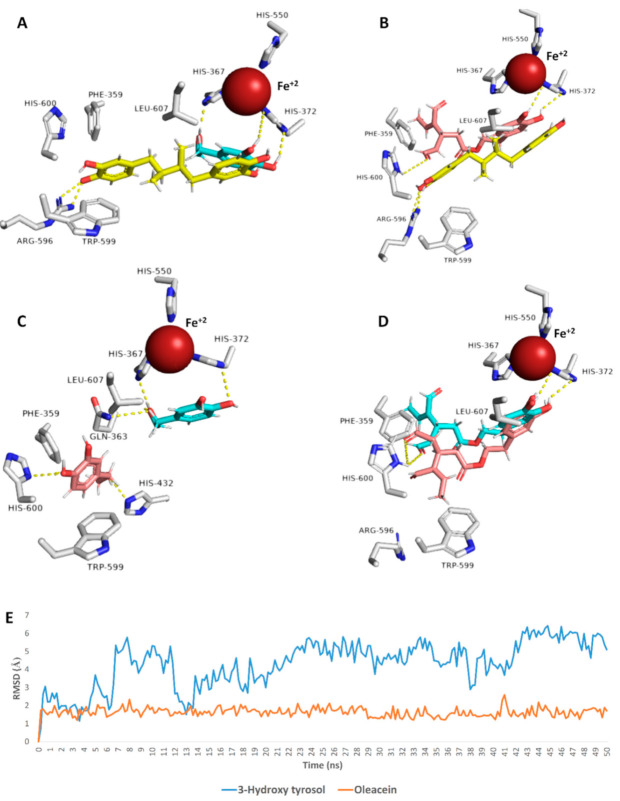
Binding modes of both HT (blue color) and OC (brick red color) in comparison with that of the co-crystalized inhibitor (yellow color) ((**A**,**B**), respectively), and their deviations throughout MDS (the blue color indicates the position at the beginning of the MDS, while the brick red color indicates the position at the end; (**C**,**D**), respectively). (**E**) The RMSDs of both HT and OC inside the 5-LOX active site during MDS, where OC was more stable than HT.

To have some insight into their 5-LOX’s mode of inhibition, we subjected both 3-hydroxy tyrosol (HT) and oleacein (OC) (**4** and **18,** respectively) to an ensemble docking protocol. Both compounds got convergent binding scores (−7.1 and −8.3 kcal/mol). In addition, their binding modes were comparable with that of the co-crystalized inhibitor nordihydroguaiaretic acid (NDGA) [55]. As shown in Figure 7A,B, the catechol moiety of both HT and OC took orientations near the Fe^+2^ similar to the co-crystalized inhibitor NDGA, and hence, they prevent the activation of the enzyme by the conversion of Fe^+2^ to Fe^+3^. Several previous plant polyphenols were reported to inhibit LOXs via their antioxidant properties [56]. HT was also able to form additional strong H-bonds (<2.5 Å) with HIS-367 and HIS-372, while the extended side chain of OC was involved in H-bonding with HIS-600 and hydrophobic interaction with PHE-359. Further 50 ns MDS refinement revealed that OC was more stable inside the binding site and deviated slightly from its original orientation (~RMSD = 1.3 Å), while HT was less stable (~RMSD = 4.8 Å) in comparison with OC, where it took a different orientation at the end of MDS (Figure 7). This behavior inside the 5-LOX binding cavity was reflected in the varying binding free energies (ΔG) of both compounds, where HT got a ΔG value of −4.4 kcal/mol, while OC got a lower value of −8.1 kcal/mol. On the other hand, both compounds were found to obey both Lipinski’s and Veber’s rules of drug-likeness [56,57] and hence, they represent good lead compounds for further development. Such structural and binding mode information explains the superior activity of OC as a 5-LOX inhibitor over that of HT and can be utilized in the future for the development of more optimized inhibitors. Additionally, these findings highlighted the potential of natural products as safe, effective, and readily available therapeutic alternatives for the management of gastric ulceration, which was particularly produced as a result of the long-term use of NSAIDs.

## 4. Conclusions

**Figure 8:**
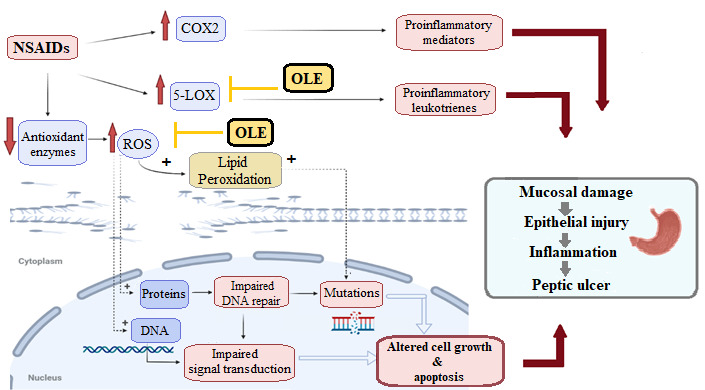
Effect of NSAIDs on both COX and 5-LOX pathways that eventually leads to peptic ulcers, and the possible protective role of OLE by decreasing the elevated oxidative stress and inhibiting 5-LOX.

The present investigation highlighted the potential gastroprotective effect of OLE against NSAIDs-induced gastritis and gastric ulcers (Figure 8). The LC-HRMS-based chemical profiling of OLE revealed the abundance of many phenolic putative compounds that can exert a profound antioxidant activity, which in turn can accelerate the ulcer healing process. To find out the most probable molecular target of these putative compounds that may also be involved in the gastro-protective effect of OLE, a comprehensive pharmacophore-based virtual screening was conducted and ended up with 5-LOX as the most possible hit. Afterwards, in vitro inhibitory testing, validated the initial virtual screening suggestion, where HT and OC showed a very potent inhibitory activity towards 5-LOX. Docking investigation, MDS, and binding free energy calculations were further shown the possible molecular interactions of each compound inside the enzyme’s active site. The present study revealed the potential of *O. europea* in providing unique lead putative compounds with potent biological activities for the management of gastric ulcers and highlighted the efficiency of the in silico based investigations in the facilitating of drug discovery and development processes. Further clinical studies are required to support our present investigation and to find out the most suitable dosage form for this valuable natural product.

## Figures and Tables

**Table 1:** H_2_O_2_ radical scavenging activity of OLE ethanolic extract.

Sample	IC_50_
OLE extract	279.2 µg/mL
Ascorbic acid	178.7 µg/mL

**Table 2:** Superoxide radical scavenging activity of OLE ethanolic extract.

Sample	IC_50_
OLE extract	419.5 µg/mL
Ascorbic acid	161.4 µg/mL

**Table 3:** Antiulcer activity of OLE.

Group	Level 1	Level II	Level III	UI (mm)	PI (%)
Normal	-	-	-	-	-
Indomethacin	22 ± 3.04	26.67 ± 2.17	14 ± 5.28	117.32 ± 23.4	-
Indomethacin + cimetidine	1.31 ± 0.32	0.3 ± 0.31	0	2.0 ± 1.01 ***	98.2
Indomethacin + total extract	2.31 ± 0.88	0	0	2.31 ± 2.17 ***	98.0

Data were represented as mean ± SD (or SEM). Significant difference between groups is analyzed by one-way ANOVA test, where *** *p* < 0.001 as compared with the control group.

## Data Availability

Data is contained within the article or supplementary material.

## Supplementary Materials

The following are available online at https://www.mdpi.com/article/10.3390/antiox10050644/s1, Figure S1: LC-HRESIMS Chromatogram of the putatively dereplicated metabolites of *Olea europaea* L. cv. Arbequina (Negative mode), Figure S2: LC-HRESIMS Chromatogram of the putatively dereplicated metabolites of *Olea europaea* L. cv. Arbequina (Positive mode), Table S1: Dereplicated metabolites from LC-HRESIMS analysis of *Olea europea* L. cv. Arbequina extract.

## References

[1] Özcan M.M., Matthäus B. (2017). A review: Benefit and bioactive properties of olive (*Olea europaea* L.) leaves. Eur. Food Res. Technol..

[2] Castejón M.L., Montoya T., Alarcón-de-la-Lastra C., Sánchez-Hidalgo M. (2020). Potential protective role exerted by secoiridoids from Olea europaea L. in cancer, cardiovascular, neurodegenerative, aging-related, and immunoinflammatory diseases. Antioxidants.

[3] Khan Y., Panchal S., Vyas N., Butani A., Kumar V. (2007). *Olea europaea*: A phyto-pharmacological review. Pharmacogn. Rev..

[4] Wang L., Geng C., Jiang L., Gong D., Liu D., Yoshimura H., Zhong L. (2008). The anti-atherosclerotic effect of olive leaf extract is related to suppressed inflammatory response in rabbits with experimental atherosclerosis. Eur. J. Nutr..

[5] Somova L., Shode F., Mipando M. (2004). Cardiotonic and antidysrhythmic effects of oleanolic and ursolic acids, methyl maslinate and uvaol. Phytomedicine.

[6] Dekanski D., Ristić S., Mitrović D. (2009). Antioxidant effect of dry olive (*Olea europaea* L.) leaf extract on ethanol-induced gastric lesions in rats. Mediterr. J. Nutr. Metab..

[7] Alirezaei M., Dezfoulian O., Sookhtehzari A., Asadian P., Khoshdel Z. (2014). Antioxidant effects of oleuropein versus oxidative stress induced by ethanol in the rat intestine. Comp. Clin. Pathol..

[8] Al-Quraishy S., Othman M.S., Dkhil M.A., Moneim A.E.A. (2017). Olive (*Olea europaea*) leaf methanolic extract prevents HCl/ethanol-induced gastritis in rats by attenuating inflammation and augmenting antioxidant enzyme activities. Biomed. Pharmacother..

[9] Xiang Z., Si J.-M., Huang H.-D. (2004). Chronic gastritis rat model and role of inducing factors. World J. Gastroenterol..

[10] Rainsford K. (1987). The effects of 5-lipoxygenase inhibitors and leukotriene antagonists on the development of gastric lesions induced by nonsteroidal anti-inflammatory drugs in mice. Agents Actions.

[11] Konturek S.J., Brzozowski T., Drozdowicz D., Beck G. (1988). Role of leukotrienes in acute gastric lesions induced by ethanol, taurocholate, aspirin, platelet-activating factor and stress in rats. Dig. Dis. Sci..

[12] Sener G., Kapucu C., Cetinel S., Cikler E., Ayanoğlu-Dülger G. (2005). Gastroprotective effect of leukotriene receptor blocker montelukast in alendronat-induced lesions of the rat gastric mucosa. Prostaglandins Leukot. Essent. Fat. Acids.

[13] Dengiz G.O., Odabasoglu F., Halici Z., Cadirci E., Suleyman H. (2007). Gastroprotective and antioxidant effects of montelukast on indomethacin-induced gastric ulcer in rats. J. Pharmacol. Sci..

[14] Mahdavi F.S., Mardi P., Mahdavi S.S., Kamalinejad M., Hashemi S.A., Khodaii Z., Mehrabani-Natanzi M. (2020). Therapeutic and Preventive Effects of Olea europaea Extract on Indomethacin-Induced Small Intestinal Injury Model in Rats. Evid. Based Complement. Altern. Med..

[15] Althaiban M. (2018). Antiulcer potential of olive leaves extract in gastric ulcer induced by indomethacin in male rats: Antioxidant and anti-inflammatory effects. Pharmacophore.

[16] Motawea M.H., Abd Elmaksoud H.A., Elharrif M.G., Desoky A.A.E., Ibrahimi A. (2020). Evaluation of Anti-inflammatory and Antioxidant Profile of Oleuropein in Experimentally Induced Ulcerative Colitis. Int. J. Mol. Cell. Med..

[17] Hassan H.A., Allam A.E., Abu-Baih D.H., Mohamed M.F., Abdelmohsen U.R., Shimizu K., Desoukey S.Y., Hayallah A.M., Elrehany M.A., Mohamed K.M. (2020). Isolation and characterization of novel acetylcholinesterase inhibitors from *Ficus benghalensis* L. leaves. Rsc. Adv..

[18] Srinivasan R., Chandrasekar M., Nanjan M., Suresh B. (2007). Antioxidant activity of *Caesalpinia digyna* root. J. Ethnopharmacol..

[19] Gürsan N. (2005). Effects of *Momordica charantia* L.(Cucurbitaceae) on indomethacin-induced ulcer model in rats. Turk. J. Gastroenterol. Off. J. Turk. Soc. Gastroenterol..

[20] Arun M., Asha V. (2008). Gastroprotective effect of *Dodonaea viscosa* on various experimental ulcer models. J. Ethnopharmacol..

[21] Batista L.M., De Almeida A.B.A., de Pietro Magri L., Toma W., Calvo T.R., Vilegas W., Brito A.R.M.S. (2004). Gastric Antiulcer Activity of *Syngonanthus arthrotrichus* SILVEIRA. Biol. Pharm. Bull..

[22] Inas Z., Hala A., Gehan H.H. (2011). Gastroprotective effect of Cordia myxa L. fruit extract against indomethacin-induced gastric ulceration in rats. Life Sci. J..

[23] Keiser M.J., Roth B.L., Armbruster B.N., Ernsberger P., Irwin J.J., Shoichet B.K. (2007). Relating protein pharmacology by ligand chemistry. Nat. Biotechnol..

[24] Phillips J.C., Braun R., Wang W., Gumbart J., Tajkhorshid E., Villa E., Chipot C., Skeel R.D., Kale L., Schulten K. (2005). Scalable molecular dynamics with NAMD. J. Comput. Chem..

[25] Lill M.A., Danielson M.L. (2011). Computer-aided drug design platform using PyMOL. J. Comput.-Aided Mol. Des..

[26] MacKerell A.D., Bashford D., Bellott M., Dunbrack R.L., Evanseck J.D., Field M.J., Fischer S., Gao J., Guo H., Ha S. (1998). All-atom empirical potential for molecular modeling and dynamics studies of proteins. J. Phys. Chem. B.

[27] Jo S., Kim T., Iyer V.G., Im W. (2008). CHARMM-GUI: A web-based graphical user interface for CHARMM. J. Comput. Chem..

[28] Jo S., Jiang W., Lee H.S., Roux B.T., Im W. (2013). CHARMM-GUI Ligand Binder for Absolute Binding Free Energy Calculations and Its Application.

[29] Langlois A. (2003). Extractive from Seven African Medicinal Plants. Ph.D. Thesis.

[30] Reyes F.J., Centelles J.J., Lupiáñez J.A., Cascante M. (2006). (2α, 3β)-2, 3-Dihydroxyolean-12-en-28-oic acid, a new natural triterpene from *Olea europea*, induces caspase dependent apoptosis selectively in colon adenocarcinoma cells. FEBS Lett..

[31] Nediani C., Ruzzolini J., Romani A., Calorini L. (2019). Oleuropein, a bioactive compound from *Olea europaea* L. as a potential preventive and therapeutic agent in non-communicable diseases. Antioxidants.

[32] Montedoro G., Servili M., Baldioli M., Selvaggini R., Miniati E., Macchioni A. (1993). Simple and hydrolyzable compounds in virgin olive oil. 3. Spectroscopic characterizations of the secoiridoid derivatives. J. Agric. Food Chem..

[33] Ha L.D., Hansen P.E., Duus F., Pham H.D., Nguyen L.H.D. (2012). Oliveridepsidones A–D, antioxidant depsidones from Garcinia oliveri. Magn. Reson. Chem..

[34] Mousouri E., Melliou E., Magiatis P. (2014). Isolation of megaritolactones and other bioactive metabolites from ‘megaritiki’table olives and debittering water. J. Agric. Food Chem..

[35] Hashmi M.A., Khan A., Hanif M., Farooq U., Perveen S. (2015). Traditional uses, phytochemistry, and pharmacology of Olea europaea (olive). Evid. Based Complement. Altern. Med..

[36] Hettiarachchi D., Liu Y., Fox J., Sunderland B. (2010). Western Australian sandalwood seed oil: New opportunities. Lipid Technol..

[37] Goossens J.P.A. (2012). Oleanolic acid. Phytochemistry.

[38] Paiva-Martins F., Gordon M.H. (2001). Isolation and characterization of the antioxidant component 3,4-dihydroxyphenylethyl 4-formyl-3-formylmethyl-4-hexenoate from olive (*Olea europaea*) leaves. J. Agric. Food Chem..

[39] Zhang Y.-L., Pan Q.-M., Zhang G.-J., Liang D. (2019). Study on chemical constituents of stems and leaves of Sapium discolor. Zhongguo Zhong Yao Za Zhi= Zhongguo Zhongyao Zazhi = China J. Chin. Mater. Med..

[40] Romani A., Mulinacci N., Pinelli P., Vincieri F.F., Cimato A. (1999). Polyphenolic content in five tuscany cultivars of *Olea europaea* L.. J. Agric. Food Chem..

[41] Pieroni A., Heimler D., Pieters L., Van Poel B., Vlietinck A. (1996). In vitro anti-complementary activity of flavonoids from oliva (*Olea europaea* L.) leaves. Pharmazie.

[42] Damak N., Allouche N., Hamdi B., Litaudon M., Damak M. (2012). New secoiridoid from olive mill wastewater. Nat. Prod. Res..

[43] Toyota M., Koyama H., Asakawa Y. (1997). Volatile components of the liverworts *Archilejeunea olivacea*, *Cheilolejeunea imbricata* and *Leptolejeunea elliptica*. Phytochemistry.

[44] Czerwińska M., Kiss A.K., Naruszewicz M. (2012). A comparison of antioxidant activities of oleuropein and its dialdehydic derivative from olive oil, oleacein. Food Chem..

[45] Pizzimenti S., Toaldo C., Pettazzoni P., Dianzani M.U., Barrera G. (2010). The “two-faced” effects of reactive oxygen species and the lipid peroxidation product 4-hydroxynonenal in the hallmarks of cancer. Cancers.

[46] Gupta P., Parminder N., Sidana J. (2012). Oxidative stress induced ulcer protected by natural antioxidant: A review. Int. Res. J. Pharm..

[47] Dekanski D., Janićijević-Hudomal S., Tadić V., Marković G., Arsić I., Mitrović D.M. (2009). Phytochemical analysis and gastroprotective activity of an olive leaf extracts. J. Serb. Chem. Soc..

[48] Zahran E.M., Abdelmohsen U.R., Hussein A.S., Salem M.A., Khalil H.E., Yehia Desoukey S., Fouad M.A., Kamel M.S. (2019). Antiulcer potential and molecular docking of flavonoids from Ocimum forskolei Benth., family Lamiaceae. Nat. Prod. Res..

[49] Wang X., Shen Y., Wang S., Li S., Zhang W., Liu X., Lai L., Pei J., Li H. (2017). PharmMapper 2017 update: A web server for potential drug target identification with a comprehensive target pharmacophore database. Nucleic Acids Res..

[50] Koscielny G., An P., Carvalho-Silva D., Cham J.A., Fumis L., Gasparyan R., Hasan S., Karamanis N., Maguire M., Papa E. (2017). Open Targets: A platform for therapeutic target identification and validation. Nucleic Acids Res..

[51] Wang L., Ma C., Wipf P., Liu H., Su W., Xie X.-Q. (2013). TargetHunter: An in silico target identification tool for predicting therapeutic potential of small organic molecules based on chemogenomic database. AAPS J..

[52] de la Puerta R., Gutierrez V.R., Hoult J.R.S. (1999). Inhibition of leukocyte 5-lipoxygenase by phenolics from virgin olive oil. Biochem. Pharmacol..

[53] Vougogiannopoulou K., Lemus C., Halabalaki M., Pergola C., Werz O., Smith A.B., Michel S., Skaltsounis L., Deguin B. (2014). One-step semisynthesis of oleacein and the determination as a 5-lipoxygenase inhibitor. J. Nat. Prod..

[54] Gilbert N.C., Gerstmeier J., Schexnaydre E.E., Börner F., Garscha U., Neau D.B., Werz O., Newcomer M.E. (2020). Structural and mechanistic insights into 5-lipoxygenase inhibition by natural products. Nat. Chem. Biol..

[55] Kemal C., Louis-Flamberg P., Krupinski-Olsen R., Shorter A.L. (1987). Reductive inactivation of soybean lipoxygenase 1 by catechols: A possible mechanism for regulation of lipoxygenase activity. Biochemistry.

[56] Lipinski C.A., Lombardo F., Dominy B.W., Feeney P.J. (1997). Experimental and computational approaches to estimate solubility and permeability in drug discovery and development settings. Adv. Drug Deliv. Rev..

[57] Veber D.F., Johnson S.R., Cheng H.-Y., Smith B.R., Ward K.W., Kopple K.D. (2002). Molecular properties that influence the oral bioavailability of drug candidates. J. Med. Chem..

