# Supplementary material for: Antiulcer Potential of Olea europea L. cv. Arbequina Leaf Extract Supported by Metabolic Profiling and Molecular Docking
Source: Antioxidants (Basel). 2021 Apr 22;10(5):644. doi: 10.3390/antiox10050644 (PMC8146603; doi:10.3390/antiox10050644)
Supplement: Supplementary file 1 [file antioxidants-10-00644-s001.zip › antioxidants-1162781-supplementary.pdf]

Supplementary Materials: Antiulcer Potential of *Olea europea* L. cv. Arbequina Leaf Extract Supported by Metabolic Profiling and Molecular Docking

Arafa Musa [1,2,*,†], Nourhan Hisham Shady [3,†], Shaimaa R. Ahmed [4,5], Taghreed S. Alnusaire [6], Ahmed M. Sayed [7], Bassam F. Alowaiesh [6,8], Ibrahim Sabouni [8], Mohammad M. Al-Sanea [9], Ehab M. Mostafa [1,2], Khayrya A. Youssif [10], Dalia H. Abu-Baih [11], Mahmoud A. Elrehany [11,12] and Usama Ramadan Abdelmohsen [3,13,*]

1 Pharmacognosy Department, College of Pharmacy, Jouf University, Sakaka, Aljouf 72341, Saudi Arabia; akmusa@ju.edu.sa
2 Department of Pharmacognosy, Faculty of Pharmacy, Al-Azhar University, Cairo 11371, Egypt
3 Department of Pharmacognosy, Faculty of Pharmacy, Deraya University, New Minia City, Minia 61111, Egypt; Norhan.shady@deraya.edu.eg
4 Department of Pharmacognosy, Faculty of Pharmacy, Cairo University, Kasr El-Aini Street, Cairo 11562, Egypt; shaimaa.ahmed@pharma.cu.edu.eg
5 Department of Pharmacognosy, College of Pharmacy, Jouf University, Sakaka, Aljouf 2014, Saudi Arabia
6 Biology Department, College of Science, Jouf University, Sakaka, Aljouf 72341, Saudi Arabia; tasalnosairi@ju.edu.sa (T.S.A.); bfalawish@ju.edu.sa (B.F.A.)
7 Department of Pharmacognosy, Faculty of Pharmacy, Nahda University, Beni-Suef 62513, Egypt; Ahmed.mohamed.sayed@nub.edu.eg
8 Olive Research Center, Jouf University, Sakaka, Aljouf 72341, Saudi Arabia; ibrahimsabouni@gmail.com
9 Pharmaceutical Chemistry Department, College of Pharmacy, Jouf University, Sakaka, Aljouf 72341, Saudi Arabia; mmalsanea@ju.edu.sa
10 Department of Pharmacognosy, Faculty of Pharmacy, Modern University for Technology and Information, Cairo 11371, Egypt; Khayrya.youssif@pharm.mti.edu.eg
11 Department of Biochemistry and molecular biology, Faculty of Pharmacy, Deraya University, New Minia City, Minia 61111, Egypt; Dalia.hamdy@deraya.edu.eg (D.H.A.-B.); Mahmoud.elrehany@deraya.edu.eg (M.A.E.)
12 Department of Biochemistry, Faculty of Medicine, Minia University, Minia 61519, Egypt
13 Department of Pharmacognosy, Faculty of Pharmacy, Minia University, Minia 61519, Egypt
* Correspondence: akmusa@ju.edu.sa (A.M.); usama.ramadan@mu.edu.eg (U.R.A.)
† Those authors have equally contributed.

Table S1. Dereplicated metabolites from LC-HRESIMS analysis of *Olea europea* L. cv. Arbequina extract.

Number	*m/z*	Name of structure	Retention time (RT)	Exact mass	Molecular formula	Peak Area
1	455.352	3-Hydroxy-12-oleanen-28-oic acid; 3β-form	11.10857	456.3592	$C_{30}H_{48}O_3$	1911.6799
2	471.34715	2,3-Dihydroxy-13(18)-oleanen-28-oic acid; (2α,3β)-form	8.92862	472.354	$C_{30}H_{48}O_4$	56903.08

3	539.17654	Oleuropein	2.8017333	540.1838	$C_{25}H_{32}O_{13}$	1975663.5
4	153.05418	2-(3,4-Dihydroxyphenyl) ethanol	2.9556967	154.0629	$C_8H_{10}O_3$	221.12705
5	507.23793	Oliverixanthone	13.451083	508.2452	$C_{30}H_{36}O_7$	1.6716347
6	153.05436	Cleroindicin F	12.28353	154.063	$C_8H_{10}O_3$	25685.429
7	553.19194	Oleuropein; 3''-Me ether	3.5709733	554.1992	$C_{26}H_{34}O_{13}$	1064936.8
8	557.223	Oleoside; 6'-O-(8-Hydroxy-2,6-dimethyl-2E-octenoyl)	2.6187433	558.2302	$C_{26}H_{38}O_{13}$	1.0510785
9	279.2321	11-Octadecen-9-ynoic acid; (E)-form	11.044853	278.224	$C_{18}H_{30}O_2$	183466.78
Number	*m/z*	Name of structure	Retention time (RT)	Exact mass	Molecular formula	Concentration
10	455.35185	3-Hydroxy-12-ursen-28-oic acid; 3β-form, 3-Ketone	10.0559	454.3445	$C_{30}H_{46}O_3$	363.18763
11	377.18056	8-Epimer, (3,4-dihydroxyphenylethyl) ester	3.3180933	378.13147	$C_{19}H_{22}O_8$	458.15578
12	439.15729	Chebulic acid 4,5-Didehydro(E-), tri-Et ester	2.9747767	438.156215	$C_{20}H_{22}O_{11}$	1.8959597
13	623.59191	Verbascoside	8.99936	624.592	$C_{29}H_{36}O_{15}$	184019.11
14	285.03958	Luteolin	4.4696567	286.047	$C_{15}H_{10}O_6$	1.2564833
15	225.11194	Olenoside A	3.0286567	226.119	$C_{11}H_{14}O_5$	115.7028
16	317.06576	Olivin	4.3482767	316.05469	$C_{17}H_{16}O_6$	1.4666315
17	203.17977	Olivacene	8.5318633	202.172	$C_{15}H_{22}$	2008631.1

18	319.11732	Oleacein	9.00507	320.1259	$C_{17}H_{20}O_6$	0.5145

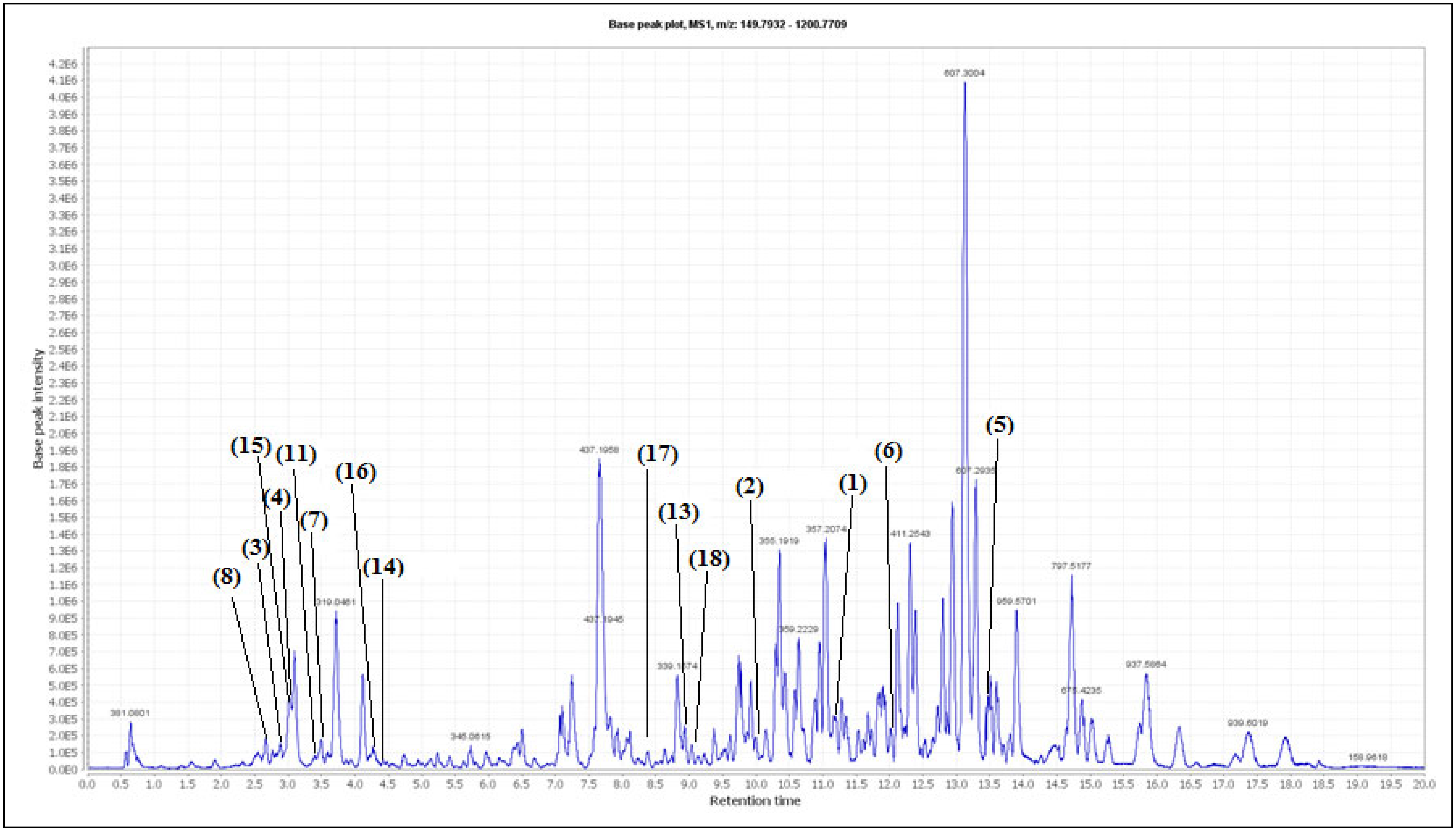

Figure S1. LC-HRESIMS Chromatogram of the putatively dereplicated metabolites of *Olea europaea* L. cv. Arbequina (Negative mode).

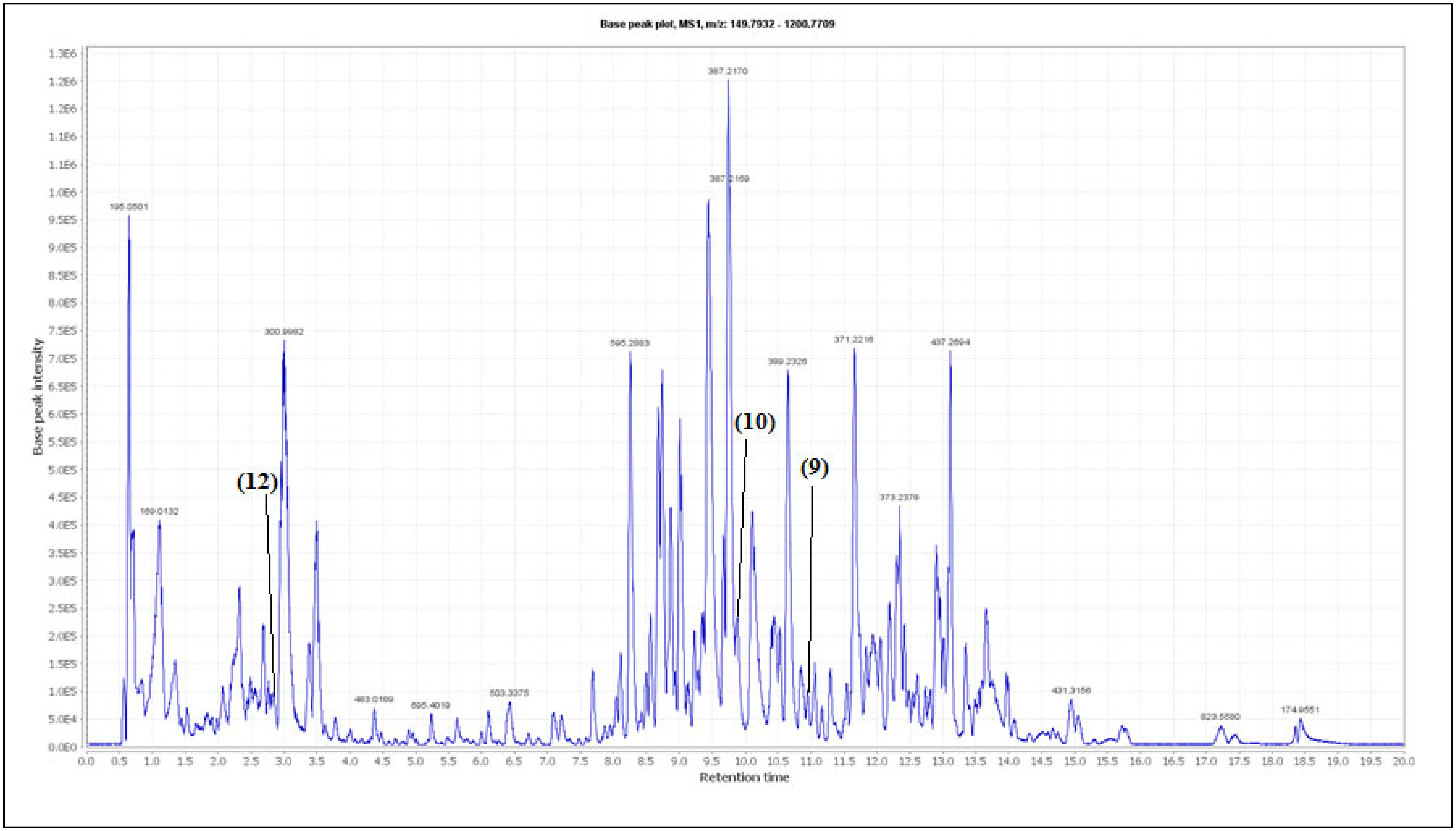

Figure S2. LC-HRESIMS Chromatogram of the putatively dereplicated metabolites of *Olea europaea* L. cv. Arbequina (Postive mode).
